# Mechanism of Photodamage of the Oxygen Evolving Mn Cluster of Photosystem II by Excessive Light Energy

**DOI:** 10.1038/s41598-017-07671-1

**Published:** 2017-08-08

**Authors:** Alonso Zavafer, Wataru Koinuma, Wah Soon Chow, Mun Hon Cheah, Hiroyuki Mino

**Affiliations:** 1 Australian National University, Research School of Biology, Canberra, Australia; 2 Division of Material Science (Physics), Graduate school of Science, Nagoya University, Furo-cho, Chikusa, Nagoya 464-8602 Japan

## Abstract

Photodamage to Photosystem II (PSII) has been attributed either to excessive excitation of photosynthetic pigments or by direct of light absorption by Mn_4_CaO_5_ cluster. Here we investigated the time course of PSII photodamage and release of Mn in PSII-enriched membranes under high light illumination at 460 nm and 660 nm. We found that the loss of PSII activity, assayed by chlorophyll fluorescence, is faster than release of Mn from the Mn_4_CaO_5_ cluster, assayed by EPR. Loss of PSII activity and Mn release was slower during illumination in the presence of exogenous electron acceptors. Recovery of PSII activity was observed, after 30 min of addition of electron donor post illumination. The same behavior was observed under 460 and 660 nm illumination, suggesting stronger correlation between excessive excitation and photodamage compared to direct light absorption by the cluster. A unified model of PSII photodamage that takes into account present and previous literature reports is presented.

## Introduction

Photosynthesis is one of the most important biochemical processes on planet Earth^1^. Photosynthesis is intrinsically a suicidal process^2^, since exposure to light will cause inhibition of the photosynthetic machinery (photoinhibition)^3^. Even though the detrimental effect of light in plants has been recognize for more than a century^4^, the ultimate causes and molecular mechanism remain unsolved. It is known that the most significant component of photoinhibition is due to the chemical changes (photodamage) of Photosystem II (PSII)^5^, which is the supramolecular complex responsible for water splitting reaction and oxygen evolution in the atmosphere.

Although there has been a great effort to solve the causes and mechanism of PSII photodamage in the last 30 years, attempts to explain PSII photodamage have been controversial. Previous reports suggested^3, 6–9^: (1) The rate constant of photoinactivation (k_PI_) of PSII is directly proportional to the irradiance in the absence of repair; (2) The time course of photoinactivation of PSII in the absence of repair is a non-reversible process that follows a first order kinetic; (3) Higher photoinactivation effect derives from shorter wavelength; (4) Photoinactivation by UV light affects two targets, the Mn_4_CaO_5_ cluster and alterations at the acceptor side of PSII.

Based on these observations, three hypotheses have been proposed. The hypothesis of PSII photodamage induced by excessive light energy absorbed by photosynthetic pigments (excessive light energy hypothesis) proposes that energy that is not used in photosynthetic reactions causes photodamage in the activity of the reaction centre^7, 8, 10^. For the excessive energy hypothesis, the damage to the PSII structure are caused by the photo-induced reactive oxygen species (ROS) or other radical species that are formed due to limitations on the donor-acceptor side or by charge recombination^7, 10^. Nevertheless, the linear relations between of the rate of photodamage (k_PI_) and irradiance reported by several groups^3, 11–14^ have been interpreted as inconsistent with excessive light energy hypothesis^12–14^ yielding an alternative hypothesis. The two-step hypothesis proposes that the direct light absorption by the Mn_4_CaO_5_ cluster causes photodamage by inducing modifications of the Mn-oxo coordination bonds in the cluster^15, 16^. This leads to the release of a Mn ion and consequent inactivation of the PSII the reaction centre^15, 16^. In this hypothesis, PSII photodamage is independent of the excessive light energy, therefore consistent with the observed linearity of k_pi_ in function of irradiance^3, 11–14^.

The third hypothesis proposes that the two-step and excessive light energy hypothesis are not mutually exclusive^6, 9, 17–19^. Each mechanism occurs preferentially depending on wavelength^9, 17^, model organism^6, 9, 17, 18^ or depth in the tissue^17, 18^.

The two step model has recently been expanded to all visible light and not only the blue regions of the visible spectrum *in vitro* ^20^. The molecular mechanism of PS II photodamage has not been clarified. Also, the fact that red and blue light were more effective in inducing inhibition on Mn_4_CaO_5_ cluster in PSII membranes suggests that PSII photodamage is dependent on the light absorption by photosynthetic pigments.

In this work, we show that the damage to the Mn_4_CaO_5_ cluster is strongly correlated to excessive light energy. By monitoring photodamage induced in PSII enriched membranes at 460 and 660 nm light, the loss of activity of Mn_4_CaO_5_cluster is ascribed to the first event of photodamage. This is not due to direct absorption of light by the cluster but to turnovers limitations in acceptor side limitations. Furthermore, Mn release is a much later consequence of PSII photodamage. The present results are discussed within the context of the two competing hypotheses of PSII photodamage. A ‘Unified Model’ of photodamage which provides satisfactory rationalisation of previous inconsistencies in literature is presented and discussed.

## Results

### Loss of PSII efficiency is faster than release of Mn^2+^ ions

**Figure 1:**
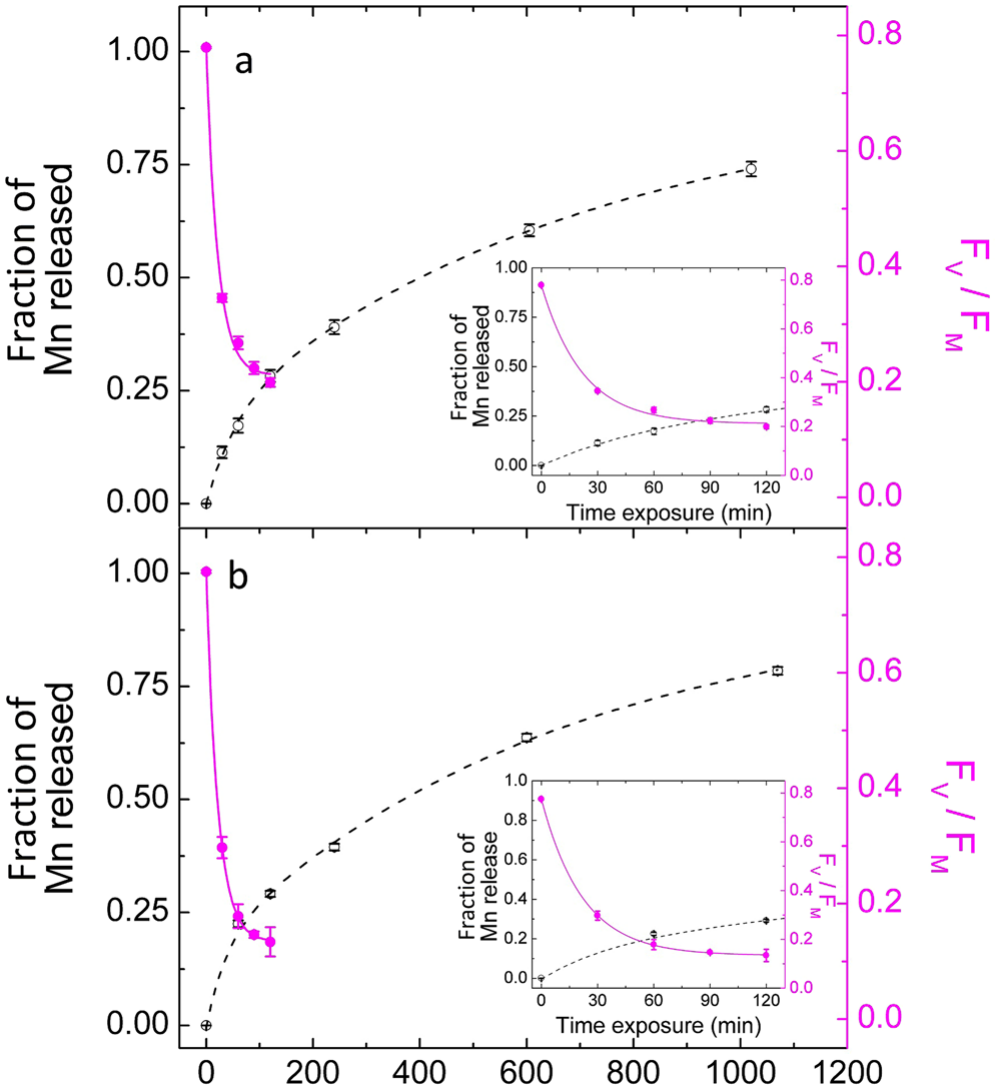
Comparison between F_V_/F_M_ ratio and the Mn^2+^ EPR signal illuminated between 460 and 660 nm. (**a**) 460 nm and (**b**) 660 nm. The black axis and line (doted) represent the normalised signal intensities of released Mn^2+^. The magenta axis and line (continuous) represent the signal intensities of F_V_/F_M_. The *inset* in each panel is a magnification of the first 120 min of illumination. Average values are presented ± standard error. For fluorescence measurements n = 6 and for EPR measurements n = 1.

**Figure 2:**
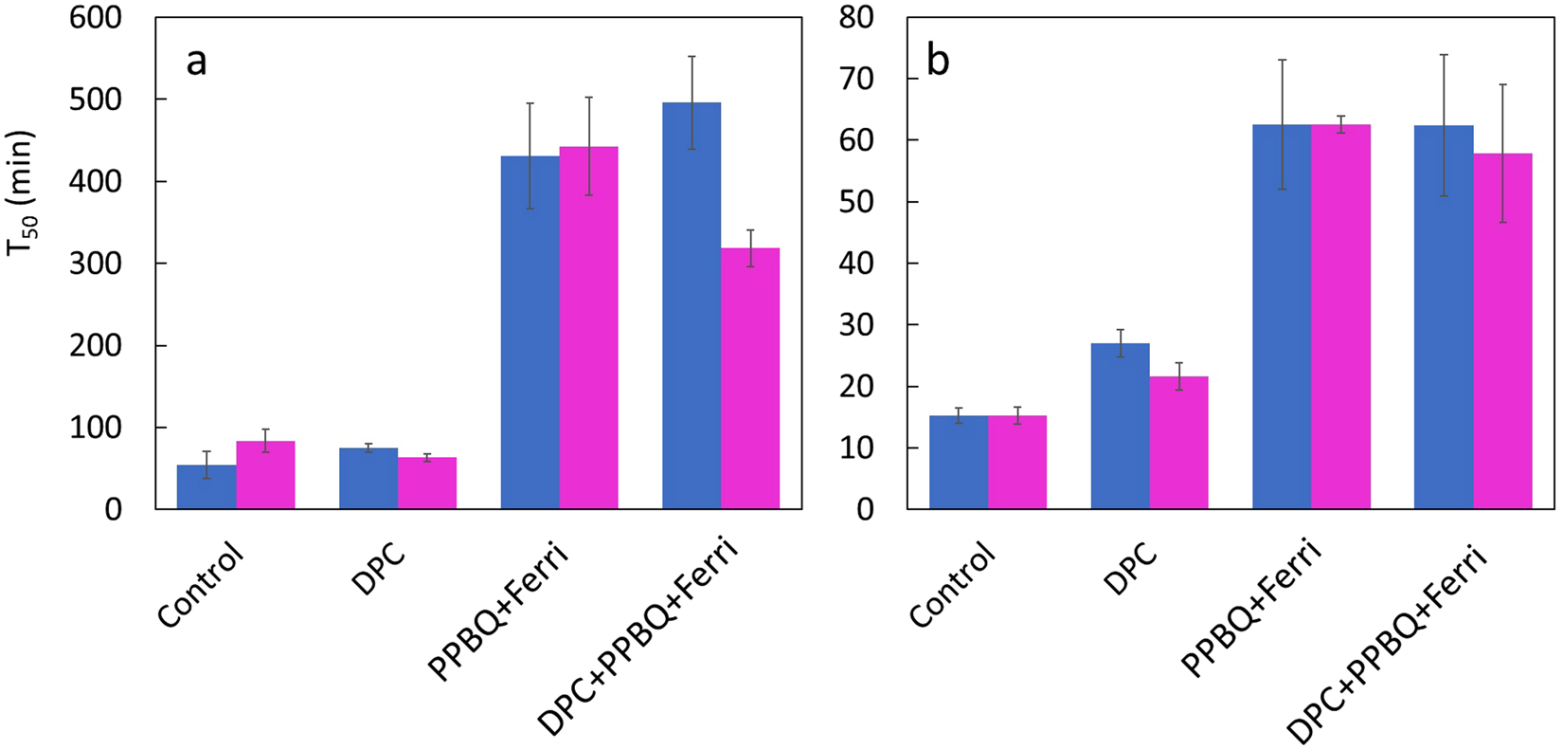
A comparison between T_50_ of loss of F_V_/F_M_ and Mn^2+^ release after illumination with 460 nm and 660 nm for 2 h. The T_50_ of (**a**) Mn^2+ ^release and (**b**) loss of F_V_/F_M_ after illumination with 460 nm (blue) and 660 nm (magenta) illuminations. Average values are presented ± standard error. For fluorescence measurements n = 6 and for EPR measurements n = 3.

Figure 1 shows the time course of the Mn^2+^ EPR signal and F_V_/F_M_ ratio detected by fluorescence of samples under 460 (panel a) and 660 nm (panel b) illumination. The increase of Mn^2+^ EPR signals are fitted with biphasic exponential functions, and decay of F_V_/F_M_ are fitted with monophasic exponential function (with an offset, y_0_) and the derived half-life times (T_50_) are presented in Table 1. There was no significant difference in the rate of release of Mn^2+^ between the two wavelengths. Closer examination between the two wavelengths at shorter time scales (Fig. 2) suggests that Mn^2+^ release is apparently faster under blue illumination. There is no noticeable difference in loss of F_V_/F_M_ between the two wavelengths. The T_50_ for decay of F_V_/F_M_ was significantly lower compared to the T_50_ of Mn^2+^ release indicating that release of Mn^2+^ occurs after loss of F_V_/F_M_. This observation shows that the release of Mn^2+^ and loss of F_V_/F_M_ are two separate events in photodamage of PSII. When PSII was fully inactivated (F_V_/F_M_ < 0.2), the Mn^2+^ released was about 25% of the total numbers (see insets in Fig. 1). This could be interpreted as, on average, one Mn ion released per molecule of PSII as a consequence of photodamage (Supplementary Figure 1 present the correlation between fluorescence measurements and oxygen evolution measurements).

**Table 1:** T_50_ values (min) for Mn^2+^ release and loss of F_V_/F_M_ during 1200 min light exposures.

		460 nm	660 nm
Mn^2+^ release	*T_fast_	38.5 ± 10.3 (0.18)	25.6 ± 14.8 (0.18)
	*T_slow_	495 ± 117 (0.73)	459.07 ± 176 (0.77)
	${y}_{0}$	0.91 ± 0.07	0.94 ± 0.12
F_V_/F_M_ decay	*T_50_	15.4 ± 1.2	15.4 ± 1.4
	${y}_{0}$	0.17 ± 0.05	0.17 ± 0.05

Each T_50_ is presented ± standard error. Mn^2+^ release was fitted to a biphasic curve and the T_50_ for the slow and fast phase are presented individually ± standard error. The ${y}_{0}$ represents the offset used for the fitting ± standard error. Amplitudes are presented in parenthesis.

### Mn^2+^ release only occurs during illumination

When the sample was kept in darkness after 120 min of illumination, there was no significant Mn^2+^ release nor uptake (Supplementary Figure 2). Release of Mn^2+^ was resumed when the samples were further illuminated after 900 min. This clearly shows that Mn^2+^ release, after the diminution of F_V_/F_M_, was caused by illumination.

### Mn^2+^ release and photodamage are caused by limitations on the acceptor side

The two-step model implies that the presence of an excess of artificial electron donor (DPC) and/or electron acceptor (PPBQ + ferricyanide) would not alter the rate of photoinactivation as it is independent of excessive excitation. In order to test this hypothesis, the first two hours of illumination at 460 and 660 nm were monitored in the absence and the presence of DPC and/or PPBQ + ferricyanide (Fig. 2). At shorter timescale (<120 min) the Mn^2+^ release data can be satisfactorily fitted to a monophasic exponential equation (Supplementary Figure 3).

At both wavelengths, the Mn^2+^ release was significantly slower in the presence of PPBQ + ferricyanide relative to the control sample (approx. 5 times see Fig. 2a). This indicates that if limitations on the acceptor side are alleviated by an artificial electron acceptor, Mn^2+^ release is suppressed. Furthermore, when PSII membranes were exposed to light in the presence of both DPC and PPBQ + ferricyanide, the rate of Mn^2+^ release also was slower (approx. 3 to 6 times see Fig. 2a). By contrast, the presence of DPC did not have any statistically significant (versus control) effect on the Mn^2+^ release during 460 nm and 660 nm illumination (Fig. 2a) nm. These results suggest that limitations on the acceptor side are the main factor in the Mn^2+^ release for wavelengths 460 and 660 nm.

Figure 2b show the corresponding F_V_/F_M_ ratios changes in the same sample. For the 460 nm illumination (Fig. 2b), in the presence of PPBQ + ferricyanide, the T_50_ for loss of F_V_/F_M_ increased compared to the control (approx. 4 times, Fig. 2b). In the presence of DPC, the T_50_ was increased relative to control (approx. 1.8 times, Fig. 2b). In the presence of PPBQ + ferricyanide and DPC, the T_50_ also increased relative to the control (approx. 4 times Fig. 2b). These results show that under 460 nm illumination there are two types of limitations, on the acceptor side and the donor side. The effect of acceptor side limitation is more pronounced than the donor side limitation, the latter being hypothesized as damage to the Mn_4_CaO_5_ cluster by direct light absorption^20^. The responses obtained at 660 nm illumination are similar to those obtained at 460 nm. Notably, there is a smaller increase in T_50_ in the ED treatment at 660 nm (Fig. 2b), indicating that limitation on the donor side at 660 nm has a lower impact on photodamage. This observation can be rationalised by the hybrid model of photodamage where an additional mechanism, viz inactivation of the Mn_4_CaO_5_ cluster by direct absorption of light at 460 nm, will cause higher level of inactivation compared to illumination at 660 nm where such contribution from such mechanism is smaller.

Figures 1 and 2 show that inactivation of PSII (loss of F_V_/F_M_) and Mn^2+^ release are strongly correlated, but they are separate processes because of the different values of T_50_. Nevertheless, at both wavelengths the acceptor side limitation had a significant impact on photoinactivation and loss of Mn^2+^. These results show that that excessive light energy does play an important role in the mechanism of PSII photodamage and the subsequent Mn^2+^ release.

### PSII efficiency is recovered if the sample is incubated with an artificial electron acceptor after photodamage

**Figure 3:**
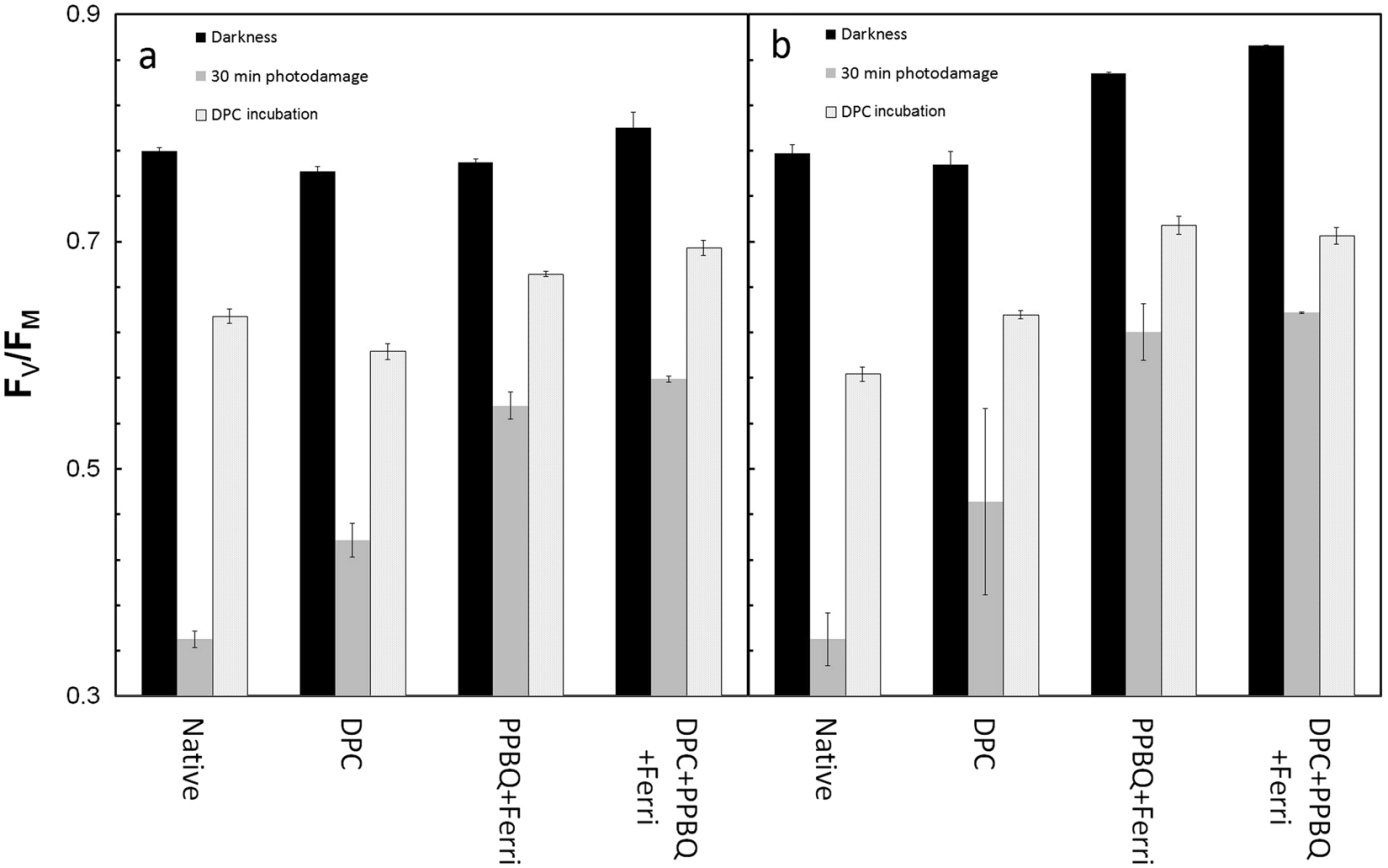
Comparison between the different treatments in the rescue experiments PSII activity in 460 nm experiments and PSII activity in the 660 nm experiments. (**a**) PSII activity in 460 nm experiments. (**b**) PSII activity in the 660 nm experiments. Average values ± standard error. For fluorescence measurements n = 6.

In order to test the effect of donor side limitation on PSII activity/efficiency, the F_V_/F_M_ ratio was measured after further addition of DPC to the illuminated samples. After illumination for 30 min and the measurement of F_V_/F_M_, the sample was incubated for 15 min in the dark in the presence of DPC and the F_V_/F_M_ was re-measured. Figure 3 shows the changes in F_V_/F_M_ ratios of the samples illuminated with 460 nm and 660 nm light, before and after addition of DPC. In all treatments, the F_V_/F_M_ ratio was recovered (≈80% relative to dark control for both wavelengths) after DPC incubation. This indicates that the Q_A_ reducing capacity (RC activity) was much less affected compared with the Mn_4_CaO_5_ cluster, in agreement with a previous report^20^. Moreover, the extent of recovery was greatest in the PPBQ + ferricyanide treated sample, further confirming the role of acceptor side limitations in photoinactivation of PSII. Additionally, this result shows that limitations on the acceptor side also cause photoinactivation of the RC, albeit to a lower extent compared to the Mn_4_CaO_5_ cluster, as a full recovery after incubation with DPC was not observed in all chemical treatments and at the two wavelengths.

## Discussion

The data presented here shows that the other PSII components (Y_z_, P680, Pheo and the Q_A_) retain high level of functionality as recovery of F_V_/F_M_ is observed after incubation with exogenous electron donor following light exposure at 460 nm and 660 nm. These observations reinforce our previous report that the primary site of PSII photodamage is at the Mn_4_CaO_5_ cluster^20^ under all visible light. This is in contrast with previous reports^21, 22^ where the Q_A_ site is proposed to be inhibited, based on results obtained by different spectroscopic methods. These discrepancies between previous results and ours can be explained by the fact that in previous works^21, 22^ the PSII activity was not measured in the presence of DPC after photodamage. In the two step mechanism^15^, it has been proposed that light induces the release of one Mn^2+^ ion from the Mn_4_CaO_5_ cluster, consequently impairing O_2_ evolving activity and PSII is inactivated. The present report shows that the rate of loss of Mn^2+^ is significantly slower compared to rate of loss of F_V_/F_M_. These results show that loss of Mn^2+^ is a consequence, not cause, of PSII photodamage and coincides with an earlier report that showed that Mn ions are released after photodamage^23^. The addition of PPBQ + ferricyanide slowed down the Mn^2+^ release, which shows that the release of Mn^2+^ can be explained by limitation on the acceptor side. Moreover, it was proposed that Mn_4_CaO_5_ cluster inactivation is independent of limitations on the acceptor side by light in the blue region^12, 16, 24^. In the present report, the hypothesis that the damage to Mn_4_CaO_5_ cluster at 460 and 660 nm is exclusively caused by directed light absorption of light by the cluster can be excluded. This is supported by the observation that the acceptor side limitation has a strong influence on the rate of photodamage since the addition of electron acceptors slowed down the loss of PSII activity. It should be noted that a minor contribution by what seems to be direct light absorption by the Mn^2+^ was observed at 460 nm; however, the effect of acceptor side limitation is much stronger in the present report. The observed lower rate of Mn^2+^ release in presence of PPBQ + ferricyanide can be satisfactorily explained by the limitation at the acceptor side as release of Mn^2+^ is a consequence of photodamage.

The hypothesis of excessive light energy absorbed by the photosynthetic pigments is a more reasonable explanation for PSII photodamage observed here. The present results are consistent with previous reported action spectra where PSII photodamage in acceptor side limited isolated thylakoids follows the pigment absorption spectra in the red region^25, 26^. In addition, loss of PSII activity and Mn^2+^ is seemingly independent of 460 and 660 nm wavelength in this study. This can be interpreted as being consistent with excessive light excitation due to pigment absorption since the apparent absorption at both 460 and 660 nm by the sample is almost the same. This argument can be extended against the two-step model where it is expected that photoinactivation at shorter wavelengths is far more effective. It should be noted that the present study does not exclude the possibility that light closer to the UV (400–420 nm) preferentially inactivates the Mn_4_CaO_5_ cluster by direct absorption^9, 15, 24, 27^. Our report is compatible with recent findings in picocyanobacteria where it was observed that direct PSII photodamage by blue photons and ROS induce damage due to excessive excitation^28^.

**Figure 4:**
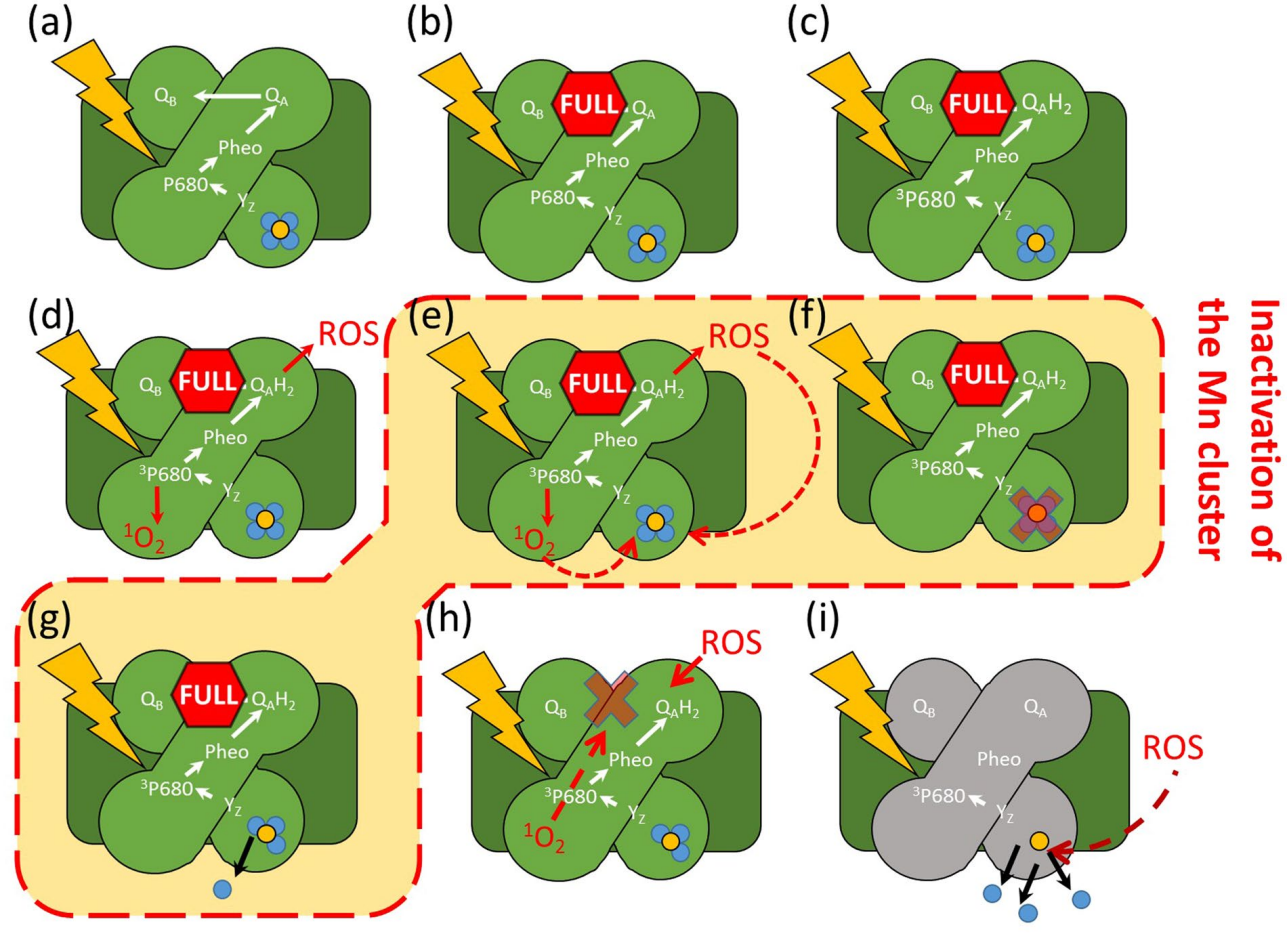
Proposed mechanism of PSII photodamage in high light. (**a**) Active PSII absorbs photons. (**b**) The pool of acceptors is fully reduced. (**c**) Repetitive turnover P680^+^Q_A_^−^ induces formation of P680 triplet or side reactions as described by Vass^8^. (**d**) Accumulation of ROS occurs at several parts of PSII and they diffuse to several sites. (**e**) Inactivation of the Mn_4_CaO_5_ cluster by ROS. (**f**) Once the Mn_4_CaO_5_ cluster is inactive, the Mn^2+^ ion is released. (**g**) In parallel to inactivation to the Mn_4_CaO_5_ cluster, ROS may deactivate the acceptor side activity on a gradual way but lower rate ROS. (**h**) As a consequence of oxidative stress the three remaining Mn^2+^ ions are released; however, at this step, PSII is completely inactive.

Based on observations presented in this report, we propose a model that explain the inactivation of the Mn_4_CaO_5_ by excessive light energy and which we termed ‘Unified Model’ for PS II photodamage summarised in Fig. 4. Damage to the PSII is primarily located at the Mn_4_CaO_5_, in agreement with the two step model. However, the primary cause of photoinactivation is attributed to limitations of the acceptor side, as per the excessive light energy hypothesis. The proposed mechanism of photodamage is as follows: By illuminating PS II in the initial state (Fig. 4a), the acceptor side is fully reduced by repetitive turnovers (Fig. 4b), leading to acceptor side limitation. By the increasing P680 triplet (and, therefore, ^1^O_2_ formation) via the recombination of P680^+^Ph^−^ and/or formation of radicals on the acceptor side (e.g. superoxide which can lead to formation of the much more reactive hydroxyl free radical), as discussed by Vass^8^, the resulting ROS attack the Mn_4_CaO_5_cluster, causing inactivation followed by release of one Mn^2+^ ion (Fig. 4c–f). By successive illumination, the acceptor side is damaged (Fig. 4d). The rest of the Mn^2+^ ions are released during a long period by ROS formed from P680 triplet or radicals from antennal chlorophylls (Fig. 4h). The model is consistent with the important role that excessive light energy absorbed by photosynthetic pigments plays an in PSII photodamage.

The double exponential kinetics observed during loss of Mn^2+^ can be explained by the following of mechanism for Mn^2+^ release: the fast phase is driven by the limitation on the acceptor side (as the addition of PPBQ + ferricyanide slowed down the release) and the slow phase represents the secondary damage to the cluster by accumulation of ROS. Once the electron transfer to Q_A_ is inhibited as all available Q_A_ is fully reduced (the electron acceptor side in PSII is full) formation of P680^+^Pheo^−^ pair still occurs and P680 triplet will be formed but at a lower rate. This results in a slow phase in which the rest of the Mn^2+^ from the cluster are released. Another possibility is that the ROS produced due to triplet chlorophyll at the antenna diffuse more slowly to the PSII inner core thus contributing to the Mn^2+^ release. This is plausible as singlet^1^O_2_ can form at antenna chlorophylls by intersystem crossing, even if PSII is inactivated.

In summary, the loss of PSII activity was faster than the fast release phase of the Mn^2+^, indicating loss of Mn from the Mn_4_CaO_5_ cluster is a consequence, not cause, of photodamage. On supplying electron acceptors, the loss of PSII activity and Mn^2+^ release was slower. On addition of electron donors after 30 min illumination the PSII activity was recovered. These results show that that Mn_4_CaO_5_ photodamage is primarily caused by limitation of the acceptor side.

## Materials and Methods

### Plant Material and sample preparation

O_2_ evolving PSII enriched membranes were prepared from fresh market spinach as described in literature^29^. The sample was suspended in a standard buffer (400 mM sucrose, 25 mM MES-NaOH, 15 mM NaCl, 5 mM MgCl_2_, pH 6.5), and adjusted to 5 mg of Chl mL^−1^. Samples were flash frozen in liquid nitrogen (LN2) and stored at −80 °C until used. The sample was kept in darkness and 4 °C at all times unless stated otherwise.

### Light irradiation of photosystem II

Before use, PSII enriched membranes were thawed in darkness and kept at 4 °C at all times in Eppendorf tubes (1.5 mL). When required, artificial electron acceptors (150 µM phenyl-p-benzoquinone (PPBQ) + 2 mM ferricyanide (Ferri)) or an artificial electron donor (300 µM diphenylcarbazide (DPC)) or a mixture of the three chemicals were added to the samples. A suspension (75 µL) of PSII enriched membranes was transferred into three custom-made EPR quartz cuvettes; the liquid was kept in a flat region of the cuvette, which had an optical thickness of 100 μm (Supplementary Figure 4). The end part of each quartz cuvette was sealed with Teflon caps and reinforced with Parafilm (Supplementary Figure 4e). Then the cuvettes were placed inside custom made LED light boxes (photoinhibition boxes) made of aluminium (Supplementary Figure 4a,d). Light was provided by arrays of 16 units of 3 W luxeon-type LEDs that were bolted into the lids of the aluminium boxes (Supplementary Figure 4a,c). The illumination was set to 1300 µmol of photons m^−2^ s^−1^ of narrow band light (460 ± 10 nm, 660 ± 10 nm see Supplementary Figure 4). In order to dissipate the heat generated by the LED array, the box lid was attached to a cooling block (Supplementary Figure 4d,g) with circulating water maintained at 4 °C; this reduced sample heating by the LED array and ensured irradiance stability of the LED sources (Supplementary Figure 4a,g). The temperature of the LED panel was 12 °C. The sample was kept at 4 °C by contact with cooling water (Supplementary Figure 4g). To enhance effective light absorption, a visible light mirror was placed under the cuvettes (Supplementary Figure 4g). The cuvettes were constantly flipped from one side to the other to ensure homogeneous illumination. The light dose (illumination time of exposure) depended on the type of experiment (see figure description). The light intensity was measured using a spectroradiometer (Comet-black, Stellar net, Inc). The spectral profile and irradiance of the LED is presented in Supplementary Figure 5.

### Determination of Mn^2+^ content by Continuous Wave Electron Paramagnetic Resonance (CW-EPR)

Mn^2+^ release was evaluated by the EPR signal of Mn^2+^. The EPR measurements were performed using a Bruker ESP 300E ESR spectrometer with a cavity type Bruker standard (ER4102ST). All measurements were performed at 5 °C and the temperature was controlled by a custom made gas flow system using nitrogen gas. The samples with a total volume of 50 µL were transferred into custom made capillary tubes (same as the ones described in photodamage experiments). The measurement conditions were: microwave frequency 9.60, the microwave power 64 mW, modulation frequency 100 kHz and modulation amplitude 8 G.

### PS II activity measurements

PSII activity was evaluated by the maximum photochemical efficiency of PSII (F_V_/F_M_) using Chl *a* fluorescence measured at room temperature by the fast rise of the Chl *a* fluorescence using a M-PEA fluorometer (Multichannel-Plant Efficiency Analyser 2, Hansatech Instruments^30^). After EPR measurement in darkness, fluorescence was measured in the same cell after more than 15 minutes dark adaptation. Three regions of the quartz cell were measured using the standard leaf clip provided by the manufacturer, the distance between two adjacent spots being at least 3 cm. The cell was illuminated with a 660 nm red saturating illumination (3000 μmol photons m^−2^ s^−1^) for 30 s. After the measurements, the sample was placed back into the LED photoinhibition box. All samples were manipulated under dim green LED light with an irradiance at the sample of less than 1 µmol photons m^−2^ s^−1^.

Measurements of oxygen evolution were as previously reported^20^.

### Recovery of the PSII activity rescue assays in the presence of an artificial electron acceptor

PS II membranes, exposed in the exact same conditions as described above, were illuminated for 30 min. Then the sample was transferred from the cuvette to an Eppendorf tube. The F_V_/F_M_ was measured after 15 min dark adaptation, by transferring 5 µl of the suspension onto paper filter (Watman 2). Then the sample was incubated with 300 µM DPC for 15 min in darkness. The activity was measured afterwards using the same method as described above.

### Data analysis

Data was analysed statistically using OriginPro software (v 9.1) and Igor Pro software (Wavemetrics). Mn^2+^ content was obtained from EPR spectra using a multicomponent fitting approach in Igor Pro, where the observed spectra were fitting with a Tyrosine D (Y_D_) - Chlorophyll and Mn^2+^ reference spectra. The 100% Mn^2+^ reference EPR spectrum was obtained by heat treatment (100 °C) of a sample of PSII enriched membrane and the 100% Y_D_-Chlorophyll reference spectrum was estimated from a pre-illuminated sample. A representative series of EPR spectra are presented in Supplementary Figure 6.

### Data availability

Data available upon request.

## Electronic supplementary material

Supplementary PDF File
