# Supplementary material for: Mechanism of Photodamage of the Oxygen Evolving Mn Cluster of Photosystem II by Excessive Light Energy
Source: Sci Rep. 2017 Aug 8;7:7604. doi: 10.1038/s41598-017-07671-1 (PMC5548768; doi:10.1038/s41598-017-07671-1)
Supplement: Supplementary file 1 — Supplementary PDF File [file 41598_2017_7671_MOESM1_ESM.pdf]

MECHANISM OF PHOTODAMAGE OF THE OXYGEN EVOLVING Mn CLUSTER OF PHOTOSYSTEM II BY EXCESSIVE LIGHT ENERGY

Alonso Zavafer[1], Wataru Koinuma[2], Wah Soon Chow[1], Mun Hon Cheah[1] , Hiroyuki Mino [2]

1 Australian National University, Research School of Biology, Canberra, Australia
2 Division of Material Science (Physics), Graduate school of Science, Nagoya University, Furo-cho, Chikusa, Nagoya, 464-8602, Japan.

Supplementary Figures.

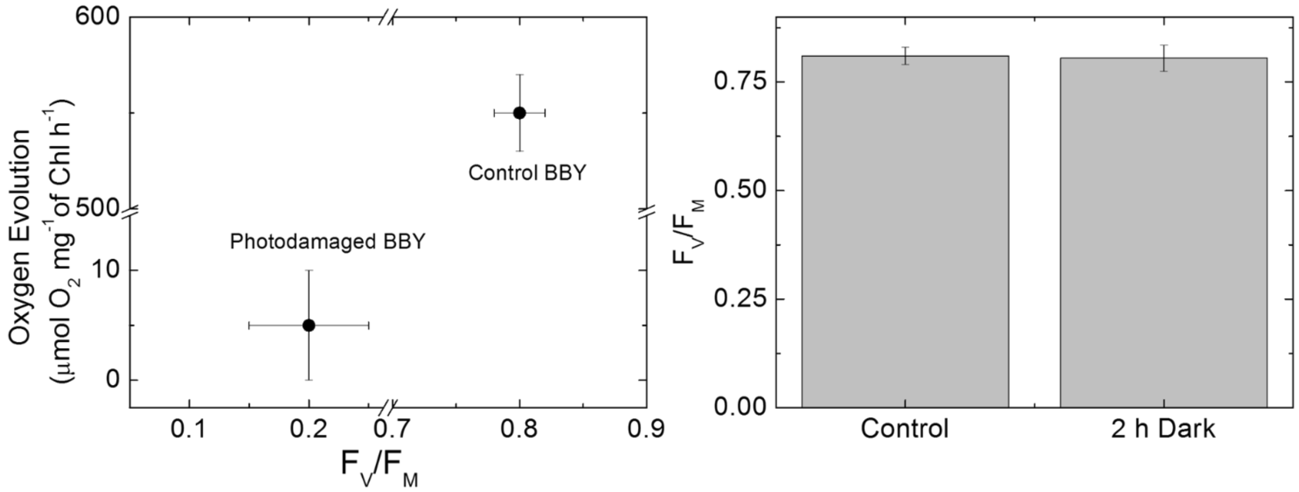

Supplementary Figure 1. Experimental controls. (a) Comparison between two techniques to measure activity of PSII in photodamage BBY and control BBY (samples kept in darkness), photodamage was induced illuminating the sample for 2h with 460 nm light. (b) PSII activity (measured by F_V/F_M) in BBY kept in the darkness for 2 h, control BBY measured at 0 h. Average values are presented ± standard deviation, n = 5.

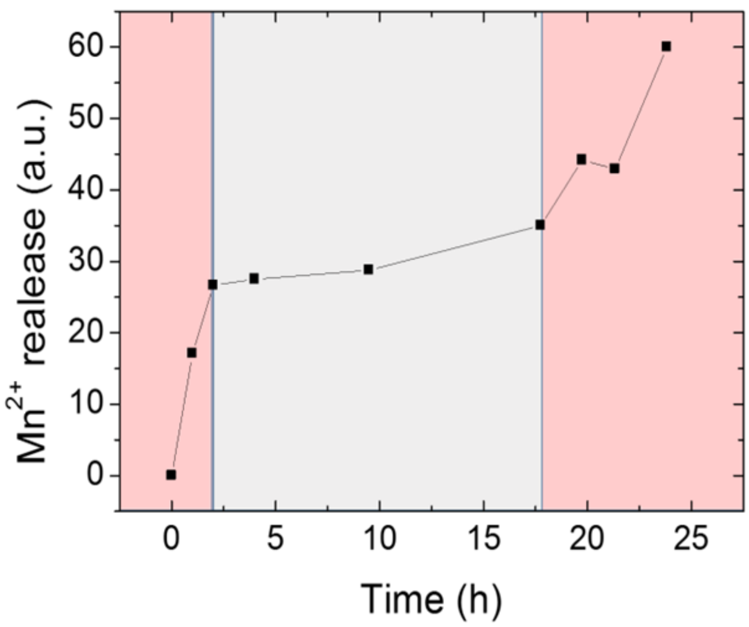

Supplementary Figure 2. Mn^{2+} release occurs only in the light A representative series of data is presented for the Mn^{2+} release (660 nm). The red frames represent illumination of the sample. The middle area represents the period when the sample was kept in darkness. n =1.

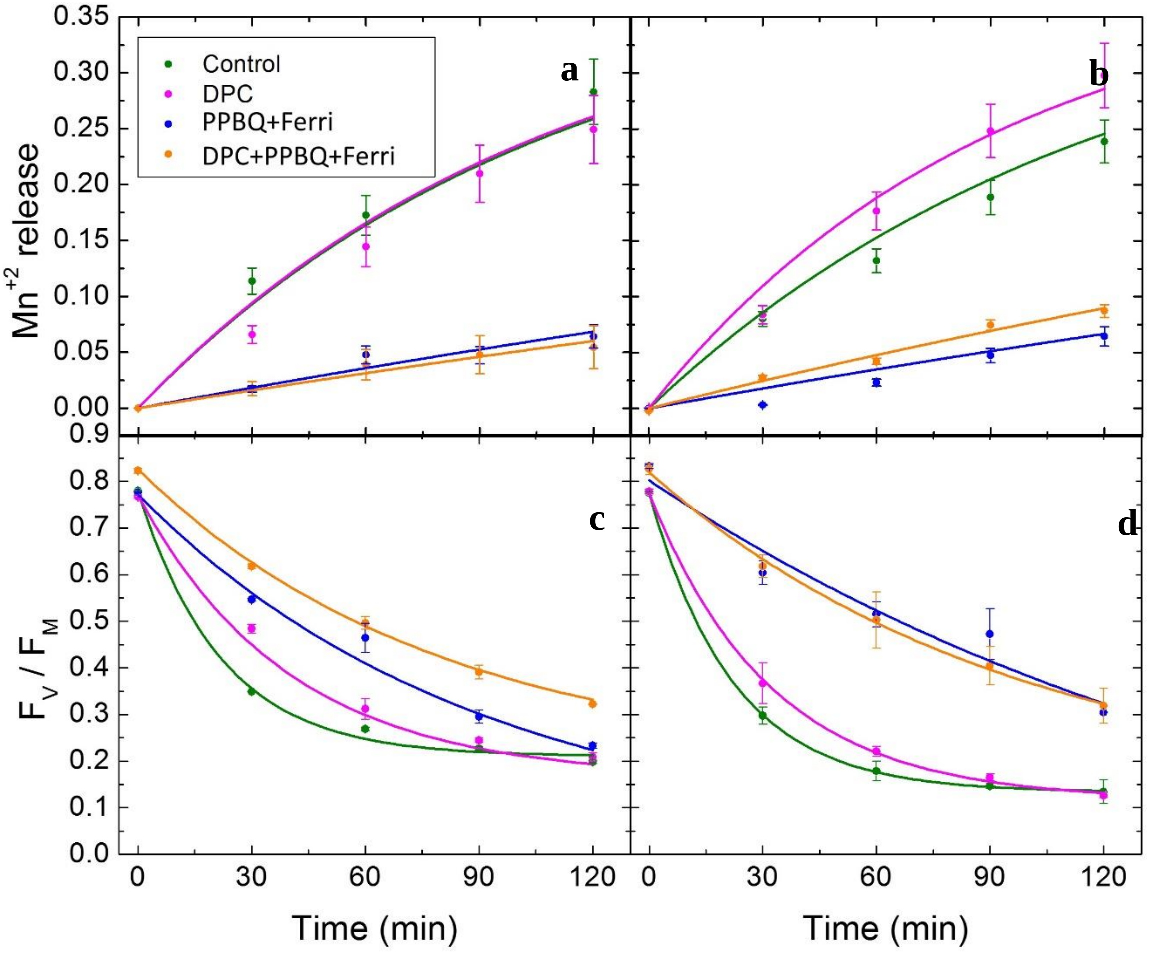

Supplementary Figure 3. Comparison between PSII photodamage and Mn^{2+} release in the different chemical treatments. (a) Mn^{2+} release during illumination at 460 nm. (b) Mn^{2+} release during illumination at 660 nm**.** In this figure, for simplicity, a single exponential fitting is used. (c) PSII photodamage at 460 nm. (d). PSII photodamage at 660 nm. Average results are presented ± std error in each case with its respective curve fitting, n = 3.

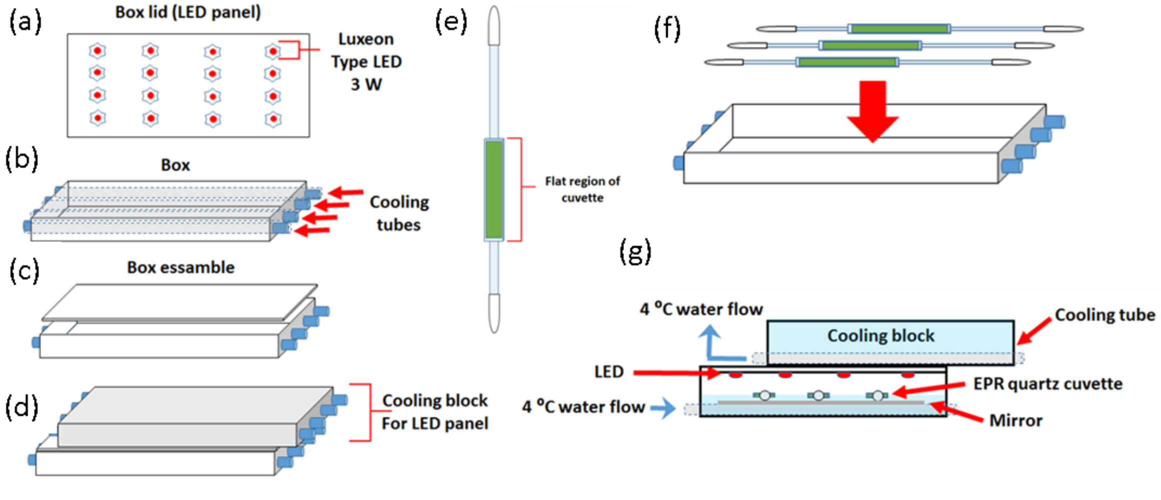

Supplementary Figure 4. Experimental setup for photodamage experiments.. (a) Diagram of the LED light source. (b) Diagram of the water cooling at the sample compartment. (c) Diagram of the box ensemble. (d) Diagram of the water cooling system for the LED. (e) Flat quartz cuvette for the EPR measurements. (f) Diagram of the cuvette samples. (g) Lateral view of the experimental setup during illumination.

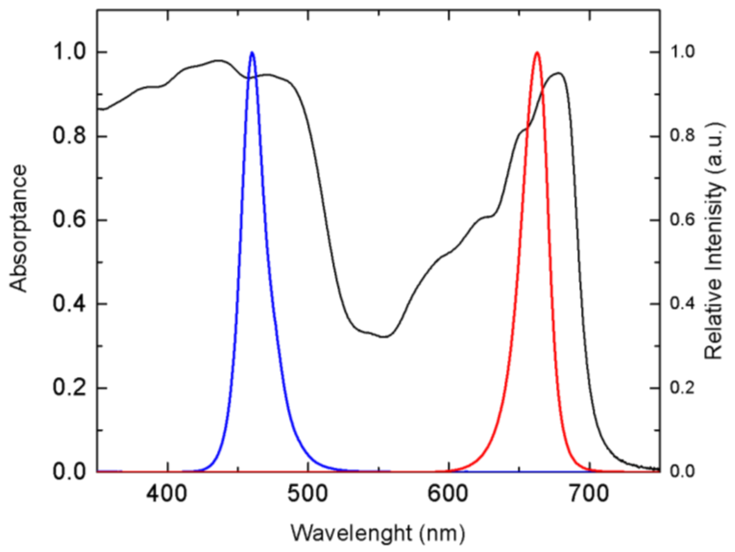

Supplementary Figure 5. Spectral profiles of the two LED light sources in respect to that of BBY-particles. BBY particles in black and LEDs of 460 (blue) and 660 (red) nm.

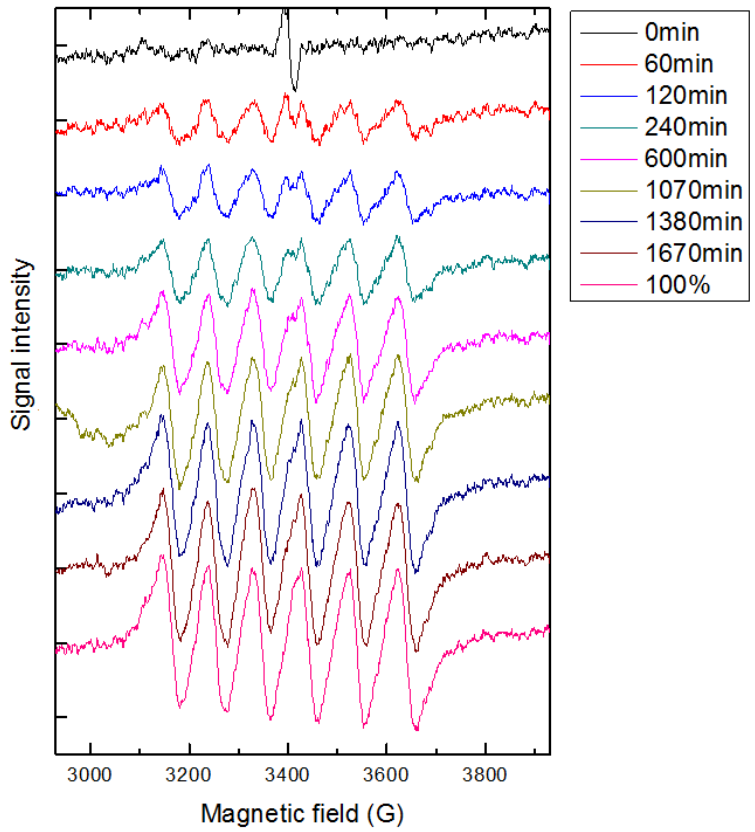

Supplementary Figure 6. EPR spectra for the Mn^{2+} signal of PSII enriched membranes after illumination. .100% of Mn^{2+} content was estimated as those in heated PS II. Only a representative set of data is presented (660 nm). Y_D signals were removed by functional processing. n = 1.

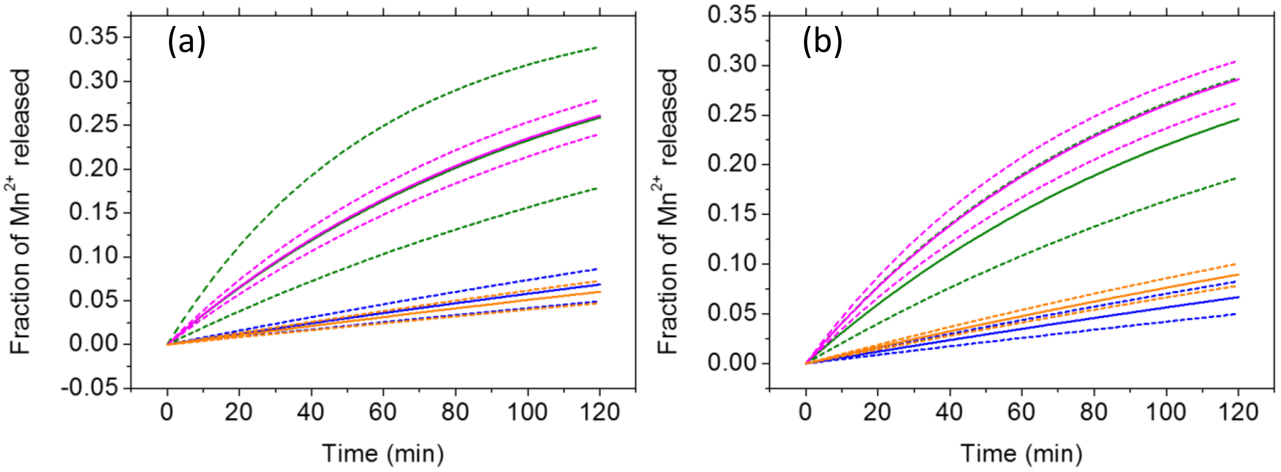

Supplementary Figure 7. Confidence region (95%) of fittings in Supplementary Figure 3 for Mn^{2+} release. Comparison between PSII photodamage and Mn^{2+} release in the different chemical treatments. Solid lines represent curves calculated based on parameters obtain from curve fitting, pointed lines represent the limits of confidence region of 95% (a) Mn^{2+} release during illumination at 460 nm. (b) Mn^{2+} release during illumination at 660 nm**.** Green: control, magenta: DPC, blue:PPBQ + Ferri, orange: DPC + PPBQ + Ferri.

Supplementary Table

Supplementary Table 1. T_{50} values (min) for Mn^{2+} release and loss of F_V/F_M during 120 min light exposures. Each T_{50} is presented ± standard error. Data was fitted to a single exponential decay A y_0 of 0.39 represents the offset used for the fitting.

	Mn2+ release		Loss of FV/FM	
	460 nm	660 nm	460 nm	660 nm
Control	54.02 ± 16.67	83.61 ± 14.36	15.25 ± 1.20	15.25 ± 1.34
DPC	75.12 ± 5.14	63.04 ± 4.77	27.03 ± 2.24	21.57 ± 2.19
PPBQ+FeCN	430.77 ± 64.52	442.88 ± 59.71	62.58 ± 10.51	62.53 ± 1.34
DPC+PPBQ+FeCN	496.03 ± 56.79	318.37 ± 22.52	62.40 ± 11.44	57.84 ± 11.19
